# Baseline heart rate variability predicts placebo hypoalgesia in men, but not women

**DOI:** 10.3389/fpain.2023.1213848

**Published:** 2023-09-20

**Authors:** Joy Krecké, Angelika M. Dierolf, Katharina M. Rischer, Fernand Anton, Marian van der Meulen

**Affiliations:** Department of Behavioural and Cognitive Sciences, University of Luxembourg, Esch-sur-Alzette, Luxembourg

**Keywords:** cognitive pain inhibition, placebo hypoalgesia, heart rate variability, sex difference, pain

## Abstract

**Introduction:**

Placebo hypoalgesic effects vary greatly across individuals, making them challenging to control for in clinical trials and difficult to use in treatment. We investigated the potential of resting vagally-mediated heart rate variability (vmHRV) to help predict the magnitude of placebo responsiveness.

**Methods:**

In two independent studies (total *N* = 77), we administered a placebo paradigm after measuring baseline HRV. In Study I, we delivered heat pain to the forearm, on skin patches treated with “real” and “control” cream (identical inactive creams). In Study II, electrical pulses to the forearm were modulated by sham transcutaneous electrical nerve stimulation. We combined data from both studies to evaluate the relationship between vagally-mediated HRV (vmHRV) parameters and the placebo response size, while also assessing sex differences in this relationship.

**Results and Discussion:**

This revealed a positive association between vmHRV and the degree of pain relief, and this effect was driven by men. These results not only reveal new insights into the (sex-specific) mechanisms of placebo hypoalgesia, but also suggest that measuring vmHRV may be helpful in predicting placebo responsiveness. Given that placebo hypoalgesic effects contribute substantially to treatment outcomes, such a non-invasive and easily obtained predictor would be valuable in the context of personalized medicine.

## 1. Introduction

Placebo hypoalgesia, i.e., expectancy-based pain relief, significantly contributes to the efficacy of pharmacological pain treatment (1). However, the degree to which it does so varies largely across individuals. There is an urgent need for better understanding this variability, and for potential predictors of the response magnitude (2). Results from studies devoted to predicting the individual placebo effect size have been largely inconclusive and the effect was shown to be mainly modulated by cognitive constructs related to expectancy (3, 4). Only few studies have investigated the predictive value of biological markers, and the chosen predictors—genotype (5) and brain function/anatomy (6)—are invasive, time-consuming and/or expensive to obtain. Here, we present two studies investigating the potential of resting vagally-mediated heart rate variability (vmHRV) as a non-invasive and practically feasible measure to predict placebo hypoalgesia, and we explored the potentially modulating role of sex.

Resting vmHRV has been presented as an index for emotion regulation skills (7), as well as for general self-regulation and flexible adaptation to the environment (8, 9). Conceptually, a greater capacity for adaptively responding to and coping with negative environmental and situational demands may also engender a better ability to regulate pain through top-down control. Indeed, greater emotion and self-regulation capacity has been associated with more efficient pain modulation (10, 11). In addition, resting vmHRV has also been associated with performance on cognitive tasks, especially those involving inhibitory control (12, 13). In turn, better cognitive inhibition abilities have been linked with more efficient top-down pain inhibition (14–17). These arguments all point to a possible association between vmHRV and descending pain modulation and suggest that vmHRV may help to predict the magnitude of placebo hypoalgesia.

From a neuroanatomical point-of-view, the brain networks involved in the modulation of vmHRV and pain show a remarkable overlap. Both the central autonomic network (CAN), which regulates HRV (18), and the descending pain control system, which underlies placebo hypoalgesia (19), include the ventromedial prefrontal cortex (vmPFC), anterior cingulate cortex (ACC), amygdala, hypothalamus and periaqueductal gray (PAG). One common output of these overlapping prefrontal-subcortical inhibitory systems for cardiac and nociceptive control is via the vagus nerve. The high-frequency (HF) component of HRV is largely under vagal (parasympathetic) control (18, 20, 21), and vagal activation has been shown to modulate pain (22–24). In particular, one study demonstrated that vagal nerve stimulation activates several brain regions including the PFC, (hypo)thalamus and PAG (23), suggesting that the vagus nerve may have its anti-nociceptive effects through activation of descending pain inhibitory pathways. The same prefrontal vagal inhibitory system may thus support both adaptive cardiac responses to environmental demands (7, 18), and favor the generation of adaptive responses to pain.

In sum, there is both a conceptual and a neuroanatomical overlap between the systems regulating HRV and pain. We hypothesized that higher baseline resting vmHRV, as a trait index of prefrontal-subcortical inhibition (25), would be associated with greater pain relief in response to a placebo.

In view of reported sex differences in both placebo responsiveness and baseline vmHRV, as well as recent calls-to-action emphasizing the need for placebo research to include sex as a biological variable to facilitate the translation of placebo mechanisms into clinical applications (26, 27), we investigated the role of sex in the relationship between vmHRV and placebo hypoalgesia. While there are some conflicting reports of sex differences in placebo effects (27–29), a systematic review showed that there is a general tendency for men to respond stronger to placebo treatments than women, with a higher placebo hypoalgesic effect reported in men (30). Similarly, studies examining the effect of sex on resting vmHRV generally demonstrate higher vmHRV values in men across a variety of time- and frequency-domain based measures (31–34). Furthermore, it is likely that sex influences the relationship between these variables, as sex differences have been reported in associations between resting vmHRV and modulation of pain (35) and emotion regulation (36). Based on these findings, we hypothesized that men would show both higher resting vmHRV as well as a stronger placebo hypoalgesic effect when compared to women. Further, we expected that sex may potentially influence the relationship between vmHRV and the degree of pain relief, though we did not have any specific hypotheses regarding the direction of this effect.

To test our hypotheses, we combined data from two studies, using similar procedures, placebo paradigms and participant samples.

## 2. Methods

### 2.1. Participants

Participants for both studies (total *N* = 77) were healthy young volunteers recruited through advertisement at the University of Luxembourg. Study I included 36 participants (16 male/20 female, mean age: 25.75 ± 5.22 years), whereas Study II included 41 participants (18 male/23 female, mean age: 23.90 ± 4.21 years). All participants gave informed consent according to the Declaration of Helsinki before participation and were paid remuneration for their effort and time (€30 in Study I; €45 in Study II, due to the different durations). Both studies were approved by Luxembourg University's local ethics committee, and Study I was additionally approved by the Luxembourg national research ethics committee (CNER). All participants were in good health and free of any cardiovascular conditions. They were also free of acute and chronic pain, the latter as assessed with the Chronic Pain Grade (37) (one female participant in Study II reported, after participation, to be suffering from knee problems, but excluding this participant did not change the results). Participants were instructed not to consume any alcohol or pain medication the day before and the day of the test session. For female participants, we recorded whether they took contraceptives; this was the case for 14 out of 20 participants in Study I, and 9/23 in Study II. Participants were excluded if they were on any other medication that is known to influence cognitive performance, cardiovascular function, or pain perception.

### 2.2. Experimental procedure study I

Participants in Study I believed that they were taking part in a study about the effect of a known analgesic cream on brain responses to painful stimulation. Functional MRI data collected are reported elsewhere (11). Participants were invited to two experimental sessions: first a laboratory session at the University of Luxembourg, during which an ECG was recorded, and 1–2 weeks later an fMRI session at the Hôpitaux Robert Schuman in Luxembourg, during which the placebo protocol was administered. Data were collected in 2014/15, and the experimenter was always the same female.

#### 2.2.1. Placebo paradigm study I

In the second experimental session of Study I, placebo hypoalgesia was induced using a well-validated protocol (38). Thermal pain stimuli were administered to the lower forearm on two different skin patches, one treated with a “real” analgesic cream and the other with a “control” cream (in reality identical). First, participants' forearms were prepared by drawing two 4 × 4 cm squares on each arm, about 10 cm apart. One of the squares on each arm was marked in red, the other in green. The position (proximal vs. distal) of the colors was counterbalanced. Participants were told that a powerful analgesic cream containing lidocaine would be applied inside the green squares, whereas an inactive control cream would be applied inside the red squares. The colors thus helped to reinforce expectations and to remind the participant of which experimental condition was currently administered during the subsequent phases of the protocol. In fact, the two creams were identical, containing a simple non-odorous white skin moisturizer. Creams were applied using gloves. Participants were told that the analgesic cream needed about 20 min to become fully effective and would then remain active for several hours. They were warned about possible side effects and were asked about any allergies against medication beforehand, to increase credibility.

#### 2.2.2. Calibration study I

During the 20 min waiting time, participants received a few practice pain stimuli on a different skin patch that was not used during the placebo protocol, to familiarize them with the stimulation procedure and rating of stimuli. Every stimulus was rated according to its intensity and unpleasantness on 100-point computerized VAS scales. The intensity scale ranged from “no pain” to “unbearable pain” and the unpleasantness scale from “not unpleasant” to “extremely unpleasant”. The pain threshold was a rating of higher than 0. We also performed a calibration procedure to determine temperatures consistently evoking VAS pain ratings of 40, 60 and 80 on the intensity scale (referring to mild, moderate, and severe pain, respectively), to be used in the subsequent manipulation and test phases. This calibration consisted of a pseudo-random series of 16 stimuli varying in temperature between 44.5–48°C, applied to the same skin patch as the practice stimuli. The pain stimuli were of the same format as the stimuli used in the placebo protocol (see Section 2.2.4 below). Pain intensity ratings were recorded and plotted in a stimulus response curve using Excel. The target VAS ratings were manually interpolated to derive the desired stimulation temperatures.

#### 2.2.3. Manipulation and test phase study I

After the calibration phase, participants were installed in the MRI scanner and we proceeded with a manipulation phase, in which we delivered six pain stimuli on each patch of the left arm. Participants were told that all pain stimuli were at 80% of their tolerance level (i.e., VAS rating of 80), but stimuli on the “real” cream patch (placebo condition) were surreptitiously lowered to the temperature evoking a rating of only 40 on the VAS (as determined in the calibration phase). This strengthened the suggestion and expectation of pain relief. The order of placebo and control conditions was counterbalanced across participants. In the test phase, participants received 15 stimuli in both the placebo and control condition (i.e., on both the “real” and the “control” cream patch) on the right arm, all at a temperature which had evoked VAS ratings of 60 during calibration. Any difference in actual ratings between the two conditions could thus be attributed to the placebo effect. Again, the order of conditions was counterbalanced across participants.

#### 2.2.4. Pain stimulation study I

Heat pain stimuli were delivered using a 3 × 3 cm peltier thermode (Somedic, Sweden). Each pain stimulus lasted 20 s, including a 1.5-second ramp-up, 17-second plateau and 1.5-second ramp-down. Each stimulus was preceded by a variable anticipation period of 4–11 s and followed by an interval of 3–7 s. After the interval, the intensity and unpleasantness scales were presented on a monitor and participants used a button box to rate the pain stimuli. After the ratings, there was an inter-stimulus interval (ISI) of 15–25 s. During the ISI, a white fixation cross was presented on a black background. This cross turned red during anticipation, signaling to participants that pain stimulation would soon start, and remained red during pain stimulation. Administration of pain stimuli and presentation of visual stimuli were synchronized using E-prime2 (Psychology Software Tools Inc, Pittsburgh, USA).

### 2.3. Experimental procedure study II

Participants in Study II were told that we were investigating age differences in the neural effects of a transcutaneous electrical nerve stimulation (TENS) device (KRES100B, HCS Electronics, China) used for pain relief. The experimental session started with the preparation for EEG and ECG recording (±45 min) and a 5-minute baseline measurement (EEG data are not reported here). Then, participants performed a few cognitive tasks (±30 min, results not reported here), after which the placebo paradigm was administered. Participants were informed beforehand about the study aim and detailed procedure. Experimenters were all female, and data were collected in 2019/20.

#### 2.3.1. Placebo paradigm study II

In the paradigm of Study II, we delivered electrical pain stimuli to the left forearm during periods of sham TENS stimulation and control periods. Two adhesive pads were attached to the skin on either side of the pain stimulation electrode on the left forearm and were connected to the TENS device. Participants were told that the TENS stimulation was at a very high frequency which is normally not detected, but the device was actually manipulated to block any output. Participants were instructed that the device would be switched on and off at specific times during the session, to compare their pain sensitivity during TENS stimulation and in the absence of stimulation. Visual and auditory cues during the session informed the participants of whether the device was supposably turned on or off. In reality, and unbeknownst to the participants, they never received TENS stimulation at any point during the experiment.

#### 2.3.2. Calibration study II

Before starting the placebo paradigm, we calibrated the electrical pain stimuli to individually adjust the intensity for each participant. We first delivered an ascending series of pulses with stepwise increasing current, to determine the detection threshold, pain threshold and pain tolerance (“familiarization phase”). Participants evaluated the perceived intensity of each pulse on a computerized 100-point visual analogue scale (VAS). The scale was divided into four equal parts, with the first part labelled (and color coded) as “no pain” (ratings of 0–25), the second part as “mild pain” (ratings of 25–50), the third part as “moderate pain” (ratings of 50–75) and the fourth part as “severe pain” (ratings of 75–100). During the remainder of the calibration phase, we aimed to determine three target stimulation intensities, evoking consistent VAS pain intensity ratings of 37.5, 62.5 and 87.5 (corresponding to the mid-points of the mild, moderate, and severe pain parts of the intensity scale). To this purpose, we delivered a series of stimuli in the approximate range of each target intensity. An automatic algorithm either increased or decreased the current, depending on the participant's rating, until the ratings were consistently on target (i.e., within a predetermined small range around the target rating). The resulting three target stimulation intensities were used in the placebo paradigm. More details on this calibration procedure are given in the Supplementary Materials.

#### 2.3.3. Manipulation phase study II

The placebo paradigm started with a manipulation phase to raise expectations of pain relief from the TENS device. Participants received four series of five pain stimuli each, two in a placebo condition (participants believed the TENS device was turned on) and two in a control condition (TENS device turned off). The conditions were alternated, and which condition came first was counterbalanced across participants. Two different anticipation sound cues, combined with two different visual cues on the monitor, indicated whether the TENS machine was supposably on or off. Participants were told that they would receive the same moderate intensity pain stimuli throughout the entire protocol, whereas in reality, they received severely painful stimuli in the control condition and mildly painful stimuli in the placebo condition. To evaluate the pain intensity of stimuli, participants used the same 100-point VAS scale as in the calibration phase. In addition, they rated the pain unpleasantness of each stimulus on an equivalent VAS scale (ratings of 0–25 were labelled as “not unpleasant”; 25–50 as “mildly unpleasant”, 50–75 as “moderately unpleasant” and 75–100 as “severely unpleasant”).

#### 2.3.4. Test phase study II

The test phase was similar to the manipulation phase, with the main difference being that participants received the same moderately painful stimulation intensity in both the placebo and control condition. The test phase was divided into two blocks, with a short re-calibration and re-manipulation phase in between the blocks. In each block, participants received eight alternating series of five stimulations: four series in the placebo and four series in the control condition. Which condition came first was counterbalanced across participants. Within each of the two blocks, two stimuli in each condition were replaced by reinforcement stimuli, to reaffirm expectations about the efficacy of the TENS device. Accordingly, at set positions within the blocks, participants received a low-intensity stimulus in the placebo condition or a high-intensity stimulus in the control condition. VAS ratings in response to these reinforcement stimuli were not included in the analyses of the pain ratings. The total number of trials included in the analyses was thus 36 in each condition (placebo and control).

#### 2.3.5. Pain stimulation study II

Electrical stimuli were administered to the participants' left volar forearm. To prepare the skin, participants were asked to wash their arm with soap. The experimenter then treated the skin with a peeling gel and applied a hydrating non-greasy gel to be absorbed. The participant was then seated in the EEG cabin, the left arm was placed on an armrest, the remaining gel was removed and a concentric surface electrode (Wasp Electrode, Specialty Developments, UK) with 7 mm diameter and a platinum pin was attached. Electrical pain stimuli were produced using a custom-built biphasic pulse generator and were delivered through a constant current stimulus isolator (A395 World precision instruments, USA). The output was limited to ±70 V, which is significantly below the international safety limits of 500 V. Pain stimuli in the experiment consisted of a biphasic pulse train of 500 ms duration at 100 Hz, with individual pulses consisting of a 2 ms positive and 2 ms negative square wave. The inter-stimulus intervals had an average duration of 20s, and each stimulus was preceded by a 500 ms anticipation cue, presented 3.5 s before the stimulus. Two seconds after each stimulus, the intensity and unpleasantness VAS scales were presented. Participants used a computer mouse for the ratings and had unlimited time to give their ratings.

### 2.4. Electrocardiogram (ECG)

In both studies, participants were in a semi-reclined position, and cardiac activity was recorded via a 3-lead ECG following Einthoven's triangle at a 1 kHz sampling rate. In Study I, we used the MP150 system and AcqKnowledge software (Biopac Systems Inc.), and in Study II, we used an ExG box and BrainVision Recorder software (BrainProducts, Gilching, Germany) for the recording. In Study I, we recorded a 5-minute eyes-open resting state ECG, and participants were instructed to simply look at a fixation cross on a monitor. In Study II, we recorded cardiac activity for 5 min as well, of which 2.5 min with eyes open (looking at a fixation cross), and 2.5 min with eyes closed. The order of these two conditions was counterbalanced across participants. Here we only report results of the eyes open condition for the sake of comparability with Study I. Several studies have shown that beat-to-beat interval data from recordings as short as 10s are sufficient to estimate relevant HRV parameters (39, 40), and that high frequency analyses can be reliably performed for recordings of 40s (41). Moreover, vmHRV parameters are among the HRV parameters with the best performance for ultra-short-term (<5 min) recordings (42).

### 2.5. Data analysis

#### 2.5.1. Placebo effect

For both studies, we created an identical index for the individual placebo effect size, by calculating the percent change in the average VAS rating in the placebo condition of the test phase, with respect to that of the control condition, according to the following formula: [(control—placebo)/control]*100. This resulted in one placebo effect score for the intensity and one for the unpleasantness ratings (**PE-I** and **PE-U**, respectively) for each participant. Higher scores indicate a greater placebo effect (i.e., a greater reduction in pain ratings in the placebo condition with respect to the control condition). In Study II, since there were no differences in pain ratings between the two experimental blocks in the test phase, ratings from both blocks were averaged.

#### 2.5.2. Heart rate variability

ECG data of both studies were analyzed using Kubios HRV Standard Version 3.3.1 (43). Kubios applies an automatic threshold-based correction of artefacts and irregular beats in inter-beat interval (IBI) data, by comparing every RR interval value against a local average interval. The local average is obtained by median filtering the IBI time series and is thus not affected by single outliers in IBI time series. The correction is made by replacing the identified artefacts with interpolated values using a cubic spline interpolation (43). We derived one time-domain metric—the root mean square of successive differences (RMSSD) between normal heartbeats, in ms—and one frequency-domain metric, namely high frequency (HF) band power in ms^2^. HF band power (HF power) was derived using a Fast Fourier Transformation and was defined as frequencies between 0.15–0.4 Hz. These metrices were chosen as they reflect parasympathetic vagal activity (18). While both situational and personal differences may influence vmHRV measures, the RMSSD primarily reflects trait influence in resting states (25). As the body mass index (BMI) is known as a factor that may influence HRV (44), we checked for potential correlations between the HRV indices and BMI (in kg/m^2^) but found none (see Supplementary Materials).

#### 2.5.3. Statistical analyses

All statistical analyses were performed using SPSS software (version 27; IBM Corp, USA). Sex differences and differences between the two studies in placebo hypoalgesia and HRV indices were probed using one-way ANOVAs. VAS pain ratings from the placebo paradigms were analyzed using separate repeated measures ANOVAs for intensity and unpleasantness ratings, with condition (Placebo vs. Control) as within-subject variable, and including sex as between-subjects factor. For Study II, we added the additional within-subject variable experimental Block (1 vs. 2). To explore the relationship between HRV and placebo hypoalgesia, and the potentially modulating effect of sex, we performed a moderation analysis on the combined data of Study I and II, using the PROCESS v3.5 macro in SPSS (45). We applied an alpha level of.05 for all statistical tests.

## 3. Results

Table 1 presents an overview of the main variables from Study I, while data from Study II are presented in Table 2. There were no significant sex differences in HRV measures or placebo effect sizes in either study (all *F* < 2.93, all *p* > .095). When comparing HRV parameters and placebo effect sizes between the two studies, we found no difference for PE-I and PE-U (both *F* < 2.50, *p* > .118), but both RMSSD [*F*(1,75) = 12.23, *p *< .001, *η_p_^2^*^ ^= .14] and HF power [*F*(1,75) = 8.30, *p* = .005, *η_p_^2^* = .10] were significantly higher in Study II than in Study I. After removing outliers, however, the difference for HF power was no longer significant (*p* = .136).

**Table 1:** Means (*M*) and standard deviations (*SD*) of the main variables from study I.

	Male (*n* = 16)		Female (*n* = 20)	
	*M*	*SD*	*M*	*SD*
VAS intensity ratings				
Manipulation phase—control	70.81	12.04	70.62	8.37
Manipulation phase—placebo	32.76	20.48	34.69	16.55
Test phase—control	62.46	15.16	55.62	18.90
Test phase—placebo	54.79	18.16	50.66	18.11
VAS unpleasantness ratings				
Manipulation phase—control	68.06	11.46	69.89	11.59
Manipulation phase—placebo	28.76	19.69	28.74	14.74
Test phase—control	58.72	16.39	52.30	23.17
Test phase—placebo	50.17	19.98	46.23	21.50
Placebo effect size				
PE-I	13.97	14.41	9.56	16.39
PE-U	17.62	20.77	13.57	23.91
HRV				
RMSSD (ms)	40.33	19.57	34.57	20.56
HF power (ms^2^)	731.27	795.12	718.67	793.03
BMI^a^ (kg/m^2^)	23.65	4.08	23.09	4.67

PE-I, behavioral placebo effect score calculated from the pain intensity ratings in the test phase; PE-U, placebo effect from the unpleasantness ratings; HRV, heart rate variability; RMSSD, root mean square of successive differences; HF, high frequency; BMI, body mass index.

^a^ BMI data of 1 (female) participant were not available.

**Table 2:** Means (*M*) and standard deviations (*SD*) of the main variables from study II.

	Male (*n* = 18)		Female (*n* = 23)	
	*M*	*SD*	*M*	*SD*
VAS intensity ratings^a,b^				
Manipulation phase—control	78.98	14.26	79.25	10.45
Manipulation phase—placebo	30.66	17.55	37.31	13.95
Test phase—control	56.84	10.06	59.58	11.24
Test phase—placebo	52.07	11.79	56.19	10.59
VAS unpleasantness ratings^a,b^				
Manipulation phase—control	76.36	15.44	78.05	11.18
Manipulation phase—placebo	28.27	12.62	32.97	15.21
Test phase—control	53.25	12.07	56.53	11.64
Test phase—placebo	46.95	13.26	52.50	10.81
Placebo effect size^a,b^				
PE-I	8.52	13.91	5.50	7.11
PE-U	12.22	13.40	6.57	9.76
HRV				
RMSSD (ms)	51.01	19.82	61.01	32.26
HF power (ms^2^)	1,152.24	1,238.61	2,184.76	2,309.69
BMI^c^ (kg/m^2^)	23.13	3.34	21.22	2.70

PE-I, behavioral placebo effect score calculated from the pain intensity ratings; PE-U, placebo effect from the unpleasantness ratings; HRV, heart rate variability; RMSSD, root mean square of successive differences; HF, high frequency; BMI, body mass index.

^a^ These values reflect the average of block 1 and block 2, as we found no differences in VAS ratings between blocks.

^b^ One male participant only completed the first block of the placebo paradigm, and average pain ratings were based on the completed trials only.

^c^ BMI data of one female participant were not available.

### 3.1. VAS pain ratings

We analyzed VAS intensity and unpleasantness ratings for Study I and Study II separately, to assess the placebo analgesic effects for the two different paradigms used.

#### 3.1.1. VAS ratings study I

**Figure 1:**
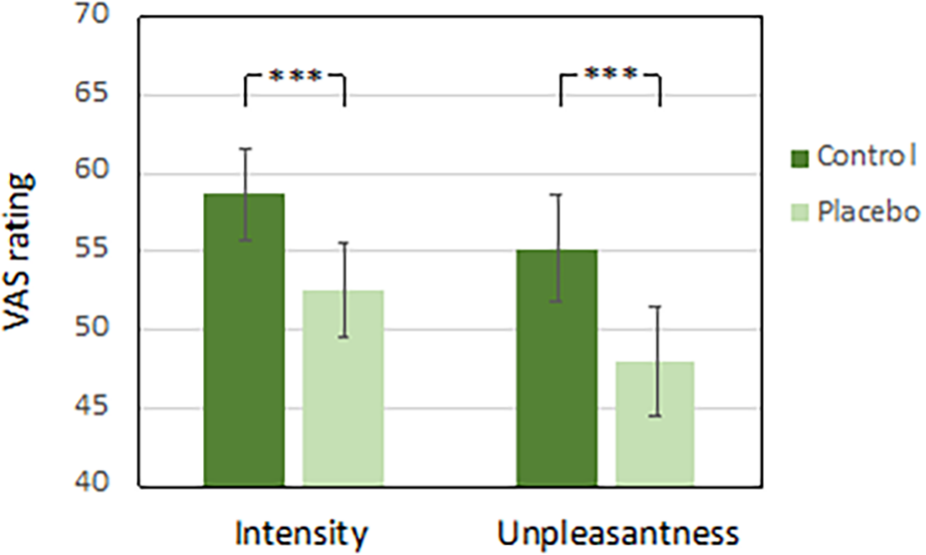
VAS pain ratings from the placebo test phase in study I. Error bars reflect the standard error of the mean. ***p < .001.

A repeated measures ANOVA on VAS intensity and unpleasantness ratings from the test phase, with condition (Placebo vs. Control) as within-subjects factor and sex as between-subjects factor revealed a significant main effect for condition, both for intensity [*F*(1, 34) = 24.85, *p* < .001, *η*_p_^2^ = .42] and unpleasantness [*F*(1, 34) = 24.13, *p* < .001, *η*_p_^2^ = .42] ratings, and no effects of sex. Overall, there was a significant reduction in subjective pain in the placebo condition compared to the control condition, indicating a robust placebo effect (see Figure 1). A similar analysis for ratings in the manipulation phase can be found in the Supplementary Materials.

#### 3.1.2. VAS ratings study II

**Figure 2:**
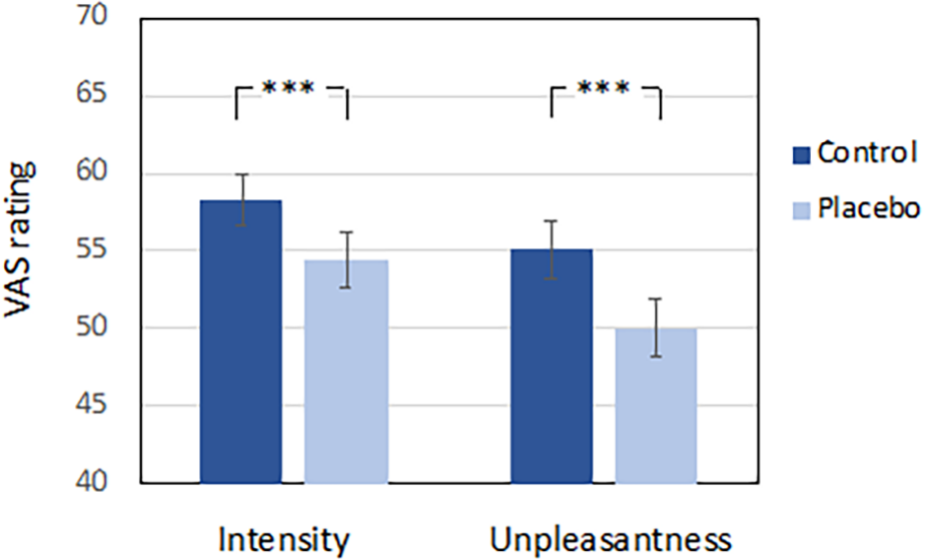
VAS pain ratings from the placebo test phase in study II. Error bars reflect the standard error of the mean. ***p < .001.

For Study II, we also performed repeated measures ANOVAs on the VAS ratings from the test phase, this time with experimental block and condition as within-subject measures and sex as between-subjects factor. For the intensity ratings, this revealed a main effect for condition [*F*(1, 38) = 21.91, *p* < .001, *η*_p_^2^ = .37], and no main effects for block or sex, nor any interactions. For the unpleasantness ratings we found the same pattern of results, with only a significant main effect for condition [*F*(1, 38) = 29.83, *p* < .001, *η*_p_^2^ = .44]. Perceived pain was significantly reduced in the placebo condition compared to in the control condition, indicating a robust placebo effect across blocks (see Figure 2). Again, the analysis of VAS ratings from the manipulation phase can be found in the Supplementary Materials.

### 3.2. Relationship between placebo hypoalgesia and vmHRV

Since we found no difference between the two studies in the placebo effect size (and despite the difference in HRV parameters), we collapsed data from both studies for further analyses. To assess the relationship between HRV and placebo hypoalgesia, and the potential influencing role of sex, we performed moderation analyses. We created four models with either PE-I or PE-U as outcome variable and either HF power or RMSSD as predictor, and with sex as moderator. Confidence intervals (95%) were computed using bootstrapping (5,000 samples). Results are summarized in Table 3. All four models explained a significant portion (between 10.2 and 13.8%) of the overall variance in the size of the placebo effect. As can be seen in Table 3, greater HRV indices significantly predicted greater placebo hypoalgesia (all *p *< .018). Sex was never a significant predictor for placebo hypoalgesia (all *p* > .055). Importantly, the interaction between HRV and sex was significant in predicting placebo hypoalgesia in all four models (all *p *< .033), indicating that sex indeed moderated the relationship between HRV and placebo hypoalgesia.

**Table 3:** Moderation by sex of the relationship between placebo hypoalgesia and HRV (study I and II collapsed).

*R* ^2^	*F*	*p*		*b* [95%CI]	SE	*t*	*p*
Model 1 (PE-I)							
.138	3.891	.012	RMSSD	.699 [.249–1.149]	.226	3.094	.003
			Sex	12.762 [–.278–25.802]	6.543	1.951	.055
			RMSSD*sex	–.357 [–.608– −.106]	.126	−2.837	.006
Model 2 (PE-I)							
.118	3.246	.027	HF power	.011 [.003–.020]	.004	2.663	.010
			Sex	1.386 [–6.282–9.055]	3.848	0.360	.720
			HF power*sex	–.006 [–.010– −.001]	.002	–2.382	.020
Model 3 (PE-U)							
.112	3.062	.033	RMSSD	.831 [.222–1.442]	.306	2.717	.008
			Sex	14.943 [–2.725–32.612]	8.865	1.686	.096
			RMSSD*sex	–.431 [–.770– −.091]	.171	–2.525	.014
Model 4 (PE-U)							
.102	2.774	.047	HF power	.014 [.003–.026]	.006	2.428	.018
			Sex	1.316 [–9.009–11.640]	5.181	.254	.800
			HF power*sex	–.007 [–.013– −.001]	.003	–2.173	.033

PE-I, behavioral placebo effect score calculated from the pain intensity ratings in the test phase; PE-U, placebo effect from the unpleasantness ratings; HF, high frequency; RMSSD, root mean square of successive differences.

**Figure 3:**
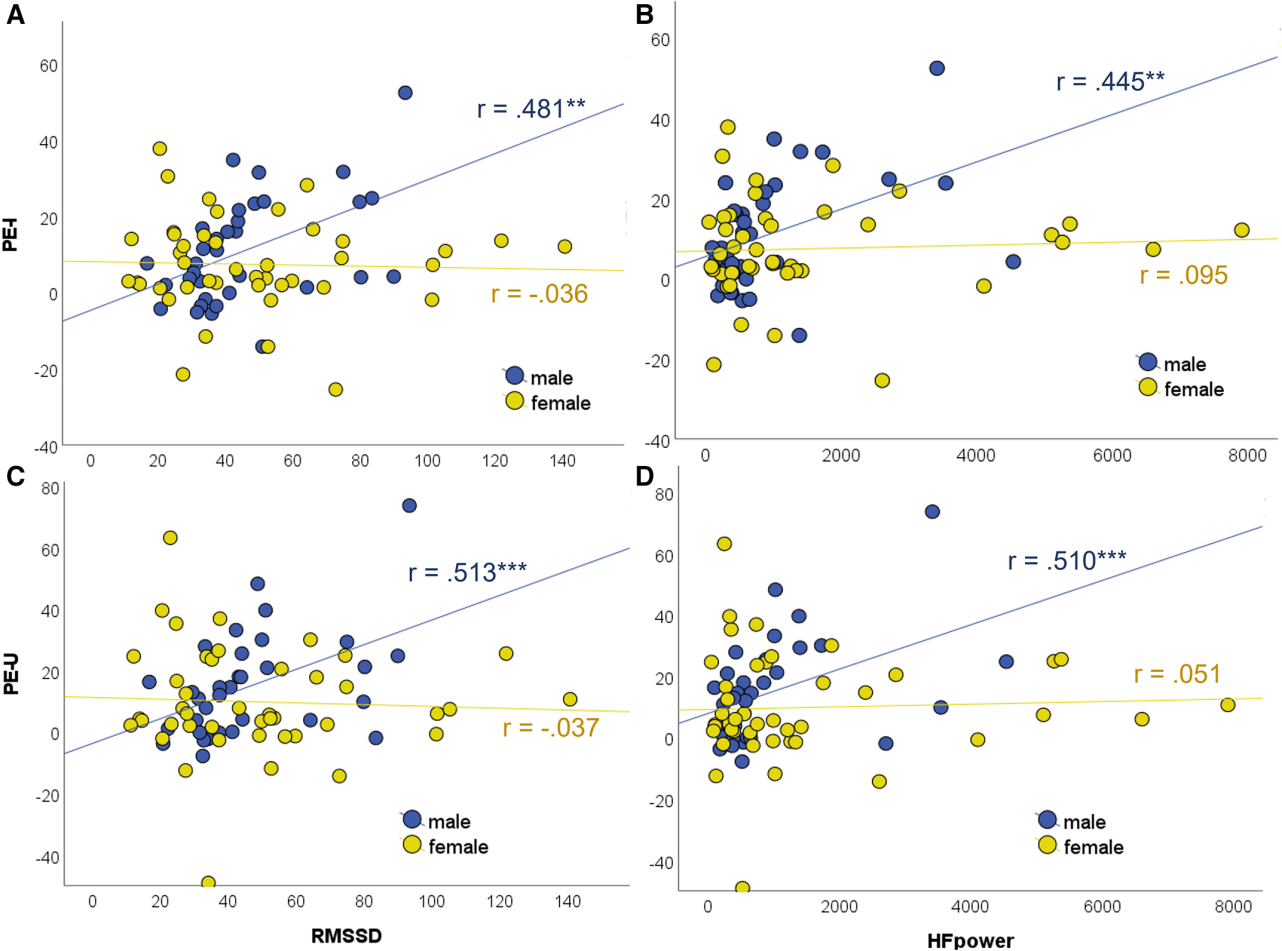
Scatterplots illustrating the moderation by sex of the association between placebo hypoalgesia and vmHRV indices (study I and II collapsed). (**A**) Moderation model 1, (**B**) Model 2, (**C**) Model 3, (**D**) Model 4 (see Table 3). Conditional effect analysis revealed significant associations for male but not for female participants (see Table 4). PE-I, behavioral placebo effect score calculated from the pain intensity ratings; PE-U, placebo effect from the unpleasantness ratings; RMSSD, root mean square of successive differences; HF, high frequency. ***p < .001, **p < .01.

Conditional effect analyses revealed that in all four models, HRV was positively associated with the placebo effect size in males, but not in female participants, for whom the association was non-significant in all four models (see Table 4). Scatterplots to illustrate this moderating effect of sex can be found in Figure 3. Note that excluding outliers did not influence the results (see Supplementary Materials).

**Table 4:** Conditional effect analysis of the moderation by sex of the relationship between placebo hypoalgesia and HRV (study I and II collapsed).

		*b* [95% CI]	SE	*t*	*p*
Model 1	Male	.342 [.126–.558]	.108	3.158	.002
	Female	–.015 [–.142–.113]	.064	–.231	.818
Model 2	Male	.006 [.002–.010]	.002	2.829	.006
	Female	<.001 [–.002–.002]	.001	.352	.726
Model 3	Male	.401 [.109–.694]	.147	2.733	.008
	Female	–.029 [–.202–.144]	.087	–.337	.737
Model 4	Male	.007 [.002–.013]	.003	2.577	.012
	Female	<.001 [–.002–.003]	.001	.311	.757

Models 1 and 2 are set up with PE-I (the behavioral placebo effect score from the intensity ratings) as dependent variable, and Models 3 and 4 with PE-U (placebo effect score from unpleasantness ratings). Models 1 and 3 are specified with RMSSD as independent variable and Models 2 and 4 with HF power (see Table 3 and Figure 3).

## 4. Discussion

### 4.1. Placebo hypoalgesia and vmHRV

Our data from two studies combined revealed that higher RMSSD and HF power, measured *before* a placebo manipulation, were associated with stronger pain reduction, confirming the expected relationship between resting vmHRV and placebo hypoalgesia. RMSSD and HF power are trait indices of effective functioning of prefrontal-subcortical inhibitory circuits (18, 21), and these same circuits are (amongst others) also involved in placebo hypoalgesia (19, 46), possibly acting through the vagus nerve (23). Individuals who effectively engage these inhibitory circuits may be able to both generate adaptive cardiac responses as well as trigger top-down modulation of nociceptive input. The positive association between vmHRV and placebo hypoalgesia in our studies is also consistent with observations that vmHRV is related to better self-regulation and adaptation to environmental challenges (8, 9, 47) and to better performance on cognitive tasks involving inhibitory control (12, 13, 48), which both in turn have been associated with more efficient top-down pain inhibition (14–17).

Several previous studies have reported associations between HRV and placebo hypoalgesia, mainly by demonstrating changes in HRV following placebo manipulations (even though not all studies on placebo hypoalgesia that assessed cardiovascular functioning have been able to observe an effect on HRV) [for an overview, see (49)]. One study, for example, demonstrated that placebo-induced increases in time-based HRV indices were related to greater pain relief (50). Baseline HRV indices, particularly HF power, have also been shown to predict lower subsequent pain ratings (51, 52). Finally, an association between higher baseline vmHRV indices and more efficient CPM was found (35), as well as a link between higher baseline RMSSD and a larger offset analgesia effect (another paradigm that assesses top-down pain control) (53). Our findings extend these results by demonstrating, for the first time, that independently measured baseline vmHRV indices can predict the magnitude of an individual's subsequent placebo hypoalgesic effect.

Interestingly, our observed association between vmHRV and placebo hypoalgesia was driven entirely by male participants. Despite general tendencies for men to show a larger placebo hypolagesic effect (30) and previous reports of higher vmHRV in men when compared to women (e.g., 31, 32), we did not find significant sex differences in these variables in our studies. However, in line with our results, several other studies have also found associations between vmHRV and pain/emotion modulation specifically in men only. The above-mentioned evidence for an association between higher baseline vmHRV indices and more efficient CPM, was found in men but not in women (35). Similarly, baseline HF power was found to be associated with pain sensitivity only in men (52). And finally, a recent study found an association between increased resting vmHRV measures and better emotion regulation (in particular self-reported use of reappraisal) only in men, not in women (36). Reappraisal is one psychological mechanism suggested to underly placebo hypoalgesia (54) and there is evidence for an association between reappraisal ability and the placebo hypoalgesia effect magnitude (11).

One potential explanation for this apparent sex-specificity of the relationship between vmHRV and placebo hypoalgesia is that men and women may recruit slightly different pain modulation mechanisms, with men activating a descending pathway that shows more overlap with the structures involved in modulating HRV, compared to women. A recent meta-analysis of neural systems underlying placebo hypoalgesia stressed the heterogeneity in placebo-related increases in brain activity and suggested that there may be different descending pain regulatory mechanisms, depending on contextual factors and participant characteristics (55). Very few studies have systematically examined sex differences in endogenous pain modulation, and results have been contradictory (56). In terms of sex differences in the neural substrate, one study demonstrated differential placebo-related modulation in the posterior insula and dorsolateral PFC in men and women (29). These findings support the hypothesis that resting HRV in women may be less predictive of the placebo effect size, because women show less overlap in the neural substrates mediating cardiac and nociceptive control. However, this remains speculative and neuroimaging studies would need to systematically investigate possible sex differences in the neural substrates of placebo hypoalgesia, which could explain the differential association with cardiac control.

Another possible reason that we found a modulation by sex of the relationship between HRV and placebo hypoalgesia is due to the influence of sex hormones. Testosterone, on the one hand, is known to have anti-nociceptive properties in both animals and humans (57–59). Importantly, in men, testosterone levels were also positively correlated with higher HF power and RMSSD (60, 61). Its ability to both decrease pain and increase HF power places testosterone in an ideal position as potential mediator of the relationship between HF power and pain modulatory mechanisms (35).

On the other hand, there is also some evidence that female sex hormones may influence both resting HRV and pain sensitivity. Both time- and frequency domain resting HRV indices, for example, have been shown to decrease across the menstrual cycle (from follicular to luteal phase) (62), and one study found a positive correlation between oestrogen levels during ovulation with both LF and HF power in healthy women (63). Likewise, experimental pain sensitivity in healthy women changes across the menstrual cycle, with generally increased sensitivity during the luteal phase relative to the follicular phase (64), again suggesting a role of sex hormones. The nature of the relationship between sex hormones, HRV and pain perception is very complex, with HRV and pain sensitivity changes observed across the menstrual cycle likely resulting from interactive effects of multiple sex hormones (62, 65). Since both our studies partly included free-cycling women (30% and 61% of female participants in Study I and II, respectively), and since we did not control for the phase in the hormonal cycle in which women participated, it is possible that hormonal fluctuations may have obscured a potential relationship between HRV and placebo hypoalgesia in our female participants.

### 4.2. Limitations and future directions

Several limitations of the presented studies should be taken into account. First of all, the individual studies both had a rather small sample size. However, by collapsing the data from both studies, we reached a sample size of 77. A post-hoc power analysis indicates that with the effect size of our moderation analysis and this combined sample size, we achieved a very high power of .89.

The use of two different placebo paradigms in Study I and II may be seen as another limitation. However, the paradigms had a very similar design, always consisting of a manipulation phase (in which expectations were raised through both verbal suggestions and surreptitiously changing pain stimulation intensities), followed by a test phase with a placebo and a control condition. Moreover, placebo effect size indices were calculated identically and there were no differences between the two studies in either of the effect size indices (PE-I and PE-U).

A last limitation, also noted above, is that the observed sex difference in the association between HRV and placebo hypoalgesia might have arisen from an interaction between sex hormones, pain and HRV. In the case of female participants, this matter is complicated further by the fact that we included both women on contraceptives as well as free-cycling women, without controlling for the phase of the hormonal cycle during which the experimental session took place. Further studies are necessary to investigate these relationships through the assessment of hormone levels and/or the careful controlling of cycle phase and contraceptive use.

## 5. Conclusion

We present data from two studies, showing a positive association between vagally mediated baseline HRV indices and subsequent pain relief, supporting the potential of vmHRV to predict placebo responsiveness, at least in men. Considering the important contribution of placebo effects to treatment outcomes (1), the ability to predict individual placebo responsiveness could guide both the selection of adequate treatment methods as well as the dosage of pharmaceutical interventions, and could enable better control of placebo effects in scientific settings, e.g., identifying placebo non-responders for clinical drug trials (66). HRV measurement is non-invasive, quick and easy to perform and to reproduce (67), further advocating its feasibility to help predict the magnitude of placebo analgesia in the clinic. However, additional studies are needed to further develop this potential (and investigate if it extends to older and patient populations, for example, chronic pain patients, who may have abnormal heart rate variability) and to explain the sex differences found.

## Data Availability

The raw data supporting the conclusions of this article will be made available by the authors, without undue reservation.
