# Supplementary material for: Baseline heart rate variability predicts placebo hypoalgesia in men, but not women
Source: Front Pain Res (Lausanne). 2023 Sep 20;4:1213848. doi: 10.3389/fpain.2023.1213848 (PMC10547887; doi:10.3389/fpain.2023.1213848)
Supplement: Supplementary file 1 [file Datasheet1.docx]

Supplementary Material

Baseline heart rate variability predicts placebo hypoalgesia in men, but not women

Krecké, Joy^*^, Dierolf, Angelika M., Rischer, Katharina, M., Anton, Fernand, van der Meulen, Marian^*^

# Methods

**1.1 Calibration procedure Study II**

In the calibration procedure, mildly (target rating: 37.5 ± 6.25), medium (target rating: 62.5 ± 6.25) and highly painful (target rating: 87.5 ± 6.25) stimuli were determined by an automatic algorithm which either increased or decreased the electrical current until the target VAS rating was reached (see Figure S1). Three cycles were completed to ensure that current-evoked ratings were consistent across time. Starting values for the first cycle were determined based on the ratings in the familiarization phase; starting values for the second and third cycle were taken from the preceding cycle. The final stimulation intensities derived from the calibration procedure were used for the placebo paradigm.


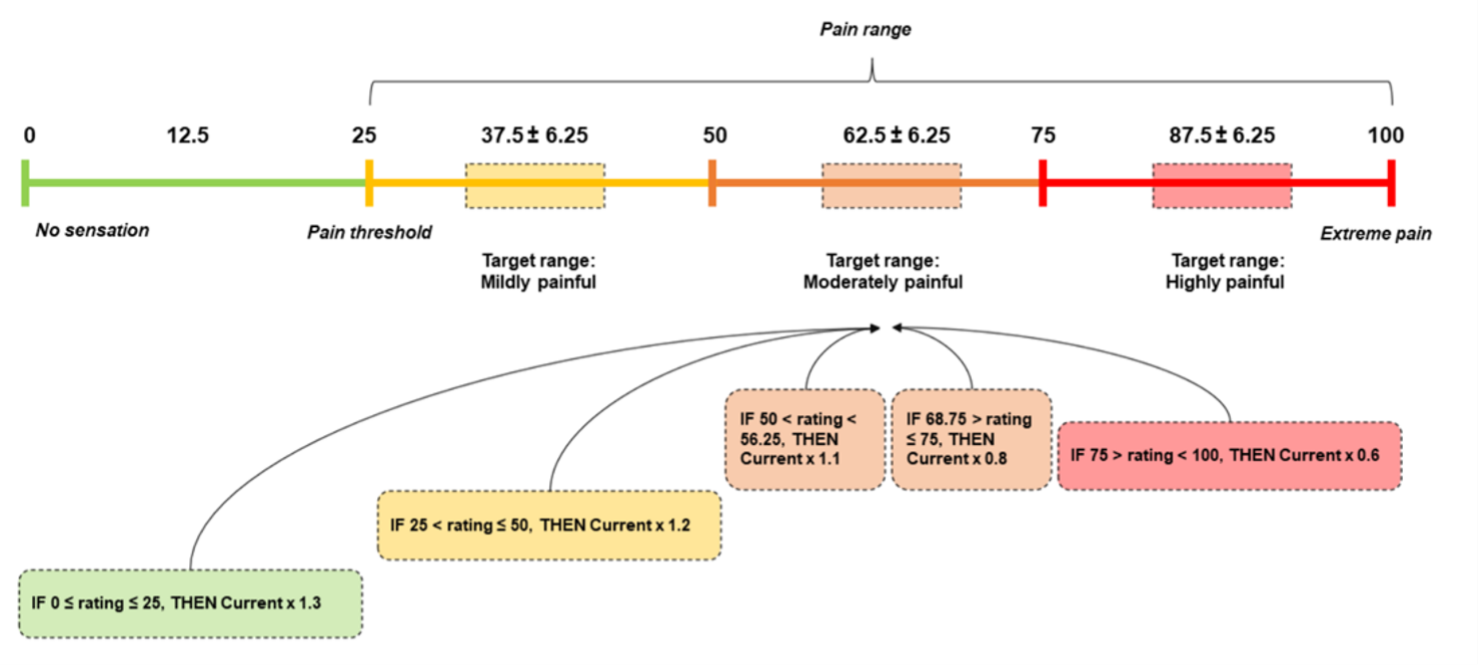


**Figure S1.** Illustration of the calibration algorithm. The current was increased or decreased by a specific factor until participants rated the current in a specified target range (colored areas on the VAS, i.e., mild, moderate, and strong pain); here depicted for the moderately painful target range. Parameters for the algorithm were determined based on pilot data.

# Results

## 2.1 Correlations between BMI and HRV indices

As the body mass index (BMI) may be inversely related to vmHRV indices (Koenig et al., 2014), we checked for potential (1-tailed Spearman) correlations between the HRV indices and BMI (in kg/m2), in both studies. In neither study, either of the HRV measures (RMSSD or HF band power) was correlated with BMI scores (Study I: both *r_s_* < -.18, both *p* > .130; Study II: both *r_s_* < .14, both *p* > .202).

## 2.2 Analysis of VAS ratings from the manipulation phase

**2.2.1 Study I**

During the manipulation phase, the temperature was lowered surreptitiously in the Placebo condition (‘real’ cream patch). We performed a repeated measures ANOVA with condition (Placebo vs. Control) as within-subjects factor and sex as between-subjects factor. For both intensity and unpleasantness ratings, there was a significant main effect for condition (*F*(1, 34) = 157.78, *p* < .001, η_p_^2^ = .82 and *F*(1, 34) = 200.42, *p* < .001, η_p_^2^ = .86, respectively). There were no effects of sex. This indicates that all participants were clearly able to distinguish between the stimuli delivered to the patch with the ‘real’ cream and those delivered to the patch with the ‘control’ cream, and confirms that the manipulation was working and expectations as to the efficacy of the cream were raised successfully.

**2.2.2 Study II**

As for Study I, we analyzed the pain intensity and unpleasantness VAS ratings from the manipulation phase, using a repeated measures ANOVA, this time with experimental block (1 vs 2) and condition (Placebo vs. Control) as within-subject measures and sex as between-subjects factor. For the intensity ratings, this revealed a significant main effect for condition (*F*(1, 38) = 262.14, *p* < .001, η_p_^2^ = .87), but not for block or sex, nor any interactions between block, sex and condition. The same was true for the unpleasantness ratings, with only a significant main effect for condition (*F*(1, 38) = 287.66, *p* < .001, η_p_^2^ = .88). Again, this validates the manipulation and confirms that positive expectations about pain relief from the TENS device were generated.

**2.2.3 Moderation analysis excluding outliers**

Outliers were identified as any values that were smaller than Q1 – 3*IQR or greater than Q3 + 3*IQR, with Q1 and Q3 referring to the first and third quartile, respectively, and IQR to the interquartile range. There were no outliers for either of the placebo effect size measures (PE-I and PE-U). However, for RMSSD, we identified one outlier (a female from Study I) and for HF power there were six outliers (one male and five females, all from Study II). Excluding these outliers had no impact on the results of the moderation analyses. In fact, the variance explained of all four models predicting placebo hypoalgesia increased slightly: **Model 1**: R^2^ = .141, *F* = 3.936, *p* = .012; **Model 2**: R^2^ = .216, *F* = 6.140, *p* < .001; **Model 3**: R^2^ = .112, *F* = 3.039, *p* = .034; and **Model 4**: R^2^ = .121, *F* = 3.083, *p* = .033. In all models, the HRV measure significantly predicted the placebo effect (all p < .009), as did the interaction term (all p < .022).

# References

Koenig J, Jarczok MN, Warth M, Ellis RJ, Bach C, Hillecke TK, Thayer JF. Body mass index is related to autonomic nervous system activity as measured by heart rate variability — A replication using short term measurements. *J. Nutr. Health Aging.* 18:300-302, 2014
